# Solvent-Driven Modulation of Shuttling Dynamics in an Autonomous Chemically Fueled Information Ratchet

**DOI:** 10.1021/acs.jpcb.5c05092

**Published:** 2025-10-15

**Authors:** Giuseppe Silvestri, Mattia P. Fossati, Federica Arrigoni, Luca Bertini, Giuseppe Zampella, Luca De Gioia, Jacopo Vertemara

**Affiliations:** Department of Biotechnology and Biosciences BtBs, 9305 University of Milano-Bicocca, Piazza dell’Ateneo Nuovo 1, Milan 20126, Italy

## Abstract

The performance of artificial molecular machines relies on the interplay between molecular design and environmental factors, yet how solvation shapes their energy landscapes and kinetics remains poorly understood. Here, we combine well-tempered and infrequent metadynamics to investigate equilibrium shuttling in a minimal [2]­rotaxane inspired by Borsley’s fuel-driven molecular motor. By systematically varying solvent polarity and hydrogen-bonding capacity, we uncover distinct thermodynamic and kinetic regimes that govern macrocycle motion. In highly polar, hydrogen-bond-accepting media, the macrocycle adopts a symmetric distribution between binding sites, with enthalpic and entropic forces in direct competition. Conversely, in low-polarity, hydrogen-bond-donating environments, the axle undergoes a conformational collapse that entropically biases occupancy toward a single station in the absence of chemical fuel. Despite comparable free-energy barriers across conditions (9–13 kcal/mol), the transition pathways exhibit pronounced solvent-dependent asymmetries and energetic ruggedness. These findings provide a molecular-level framework for understanding how solvation dictates passive ratchet behavior and offer strategic insights for designing high-performance molecular machines tailored to complex media.

## Graphical abstract

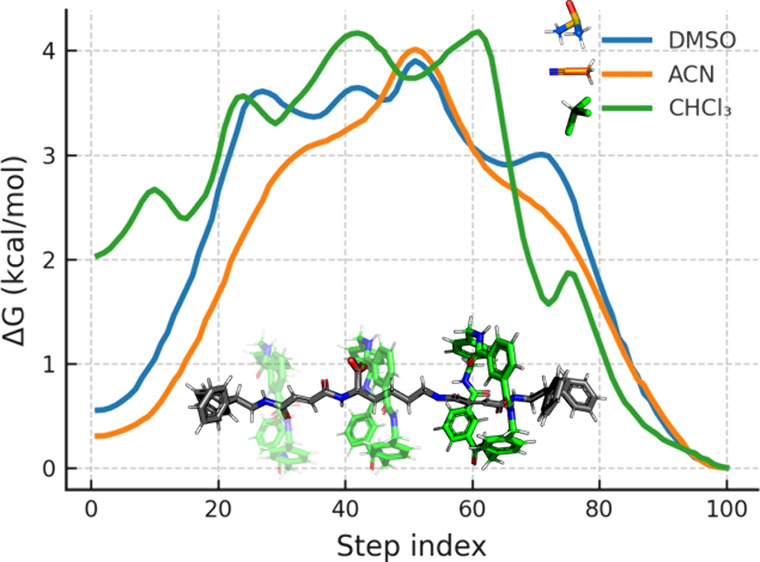

## 1 Introduction

The development of artificial molecular machines (AMMs) has opened new frontiers in mimicking complex biological functions at the nanoscale.^1–7^ Among these, rotaxanes^8,9^ have emerged as versatile components in nanoscale devices, capable of performing tasks such as controlled transport, catalysis, and molecular shuttles.^10–22^ By modulating the position of the ring along the axle, rotaxanes can operate either as bistable switches or, under continuous nonequilibrium operation, as directional motors. A particularly elegant operational principle underlying directional behavior in these systems is the information ratchet mechanism.^23–34^ In contrast to processes governed purely by thermodynamic or kinetic control, information ratchets couple stochastic conformational fluctuations with an orthogonal, energy-consuming chemical reaction. The resulting dissipative input rectifies molecular motion, biasing transitions along a reaction coordinate in a preferred direction. Directionality thus emerges not from a single energy gradient but from the interplay between thermal noise, kinetic asymmetry, and molecular recognition.

An exemplary realization of this principle is the [2]­rotaxane system developed by Borsley and co-workers.^35,36^ In this molecular motor, a macrocycle shuttles between two fumaramide recognition sites on a flexible axle, while a nearby carboxylate group catalyzes a carbodiimide-fueled transformation. The transient formation of a kinetic barrier biases the macrocycle’s motion, favoring progression in one direction over the other. This directional behavior arises from kinetic gating: rather than simply following the most thermodynamically favorable pathway, the system progresses based on differences in reaction rates at specific locations along the axle. The macrocycle’s position modulates the local chemical environment such as the accessibility or reactivity of catalytic groups, which in turn affects how likely a given chemical step is to occur. This coupling between spatial arrangement and reaction kinetics enables the system to rectify random fluctuations into net directional motion. However, such behavior cannot be fully understood without considering the role of the surrounding medium. Solvent properties (polarity, hydrogen bonding ability, steric bulk etc.) can significantly influence molecular recognition, macrocycle–axle interactions, and the accessibility of transition states.^37–39^ These solvent-dependent effects modulate both the shape of the free energy landscape and the magnitude of the kinetic barriers associated with ring translocation. For instance, polar solvents may stabilize exposed charged groups or hydrogen-bond donors/acceptors, while bulky solvents may restrict conformational freedom and introduce friction that affects the dynamics of the shuttling process. Despite their importance, a systematic understanding of how solvent properties affect information ratchet operation remains limited.

To address this gap, we present an in silico study of Borsley’s rotaxane under equilibrium conditions using classical molecular dynamics (MD) combined with enhanced sampling techniques, specifically well-tempered metadynamics^40,41^ (WT-MetaD) and its infrequent variant.^42^ Similar computational approaches have been successfully employed in previous studies to investigate the dynamics and thermodynamics of rotaxanes and other mechanically interlocked molecules (MIMs),^43–54^ even highlighting the role of the solvent and the surrounding environment.^55–63^ By isolating the thermodynamic and kinetic aspects of macrocycle shuttling in the absence of fuel, we aim to disentangle the intrinsic solvent-induced modulations from those arising from chemical reactions. Standard WT-MetaD allows us to reconstruct the free energy landscape and decompose it into enthalpic and entropic contributions,^63–65^ while infrequent WT-MetaD enables an estimation of transition rates between metastable states across different solvents. In particular, we explore how solvent identity influences the macrocycle’s distribution between the binding sites, the stability and symmetry of metastable states, and the magnitude of free-energy barriers separating them. We also explore how the macrocycle’s location, especially near the catalytic carboxylate group, affects the structure of the surrounding area. In some solvents, this setup seems to help the system adopt geometries that may make later chemical steps easier, by reducing the need for large rearrangements. These findings underline how molecular structure and solvent can work together to shape the function of fuel-driven molecular machines.

### 1.1 System Description, Function, and Goals

The system analyzed in this study is a [2]­rotaxane proposed by S. Borsley et al.,^35,36^ as shown in Figure 1(A, B). The axis has two phenyl groups at each end that act as stoppers for the ring, while two fumaramide groups are unequally located on either side of a catalytic carboxylate group, acting as binding stations for the macrocycle, a benzylic amide ring including four amide moieties. Thus, a proximal carboxylate station and a distal one are defined.

**Figure 1:**
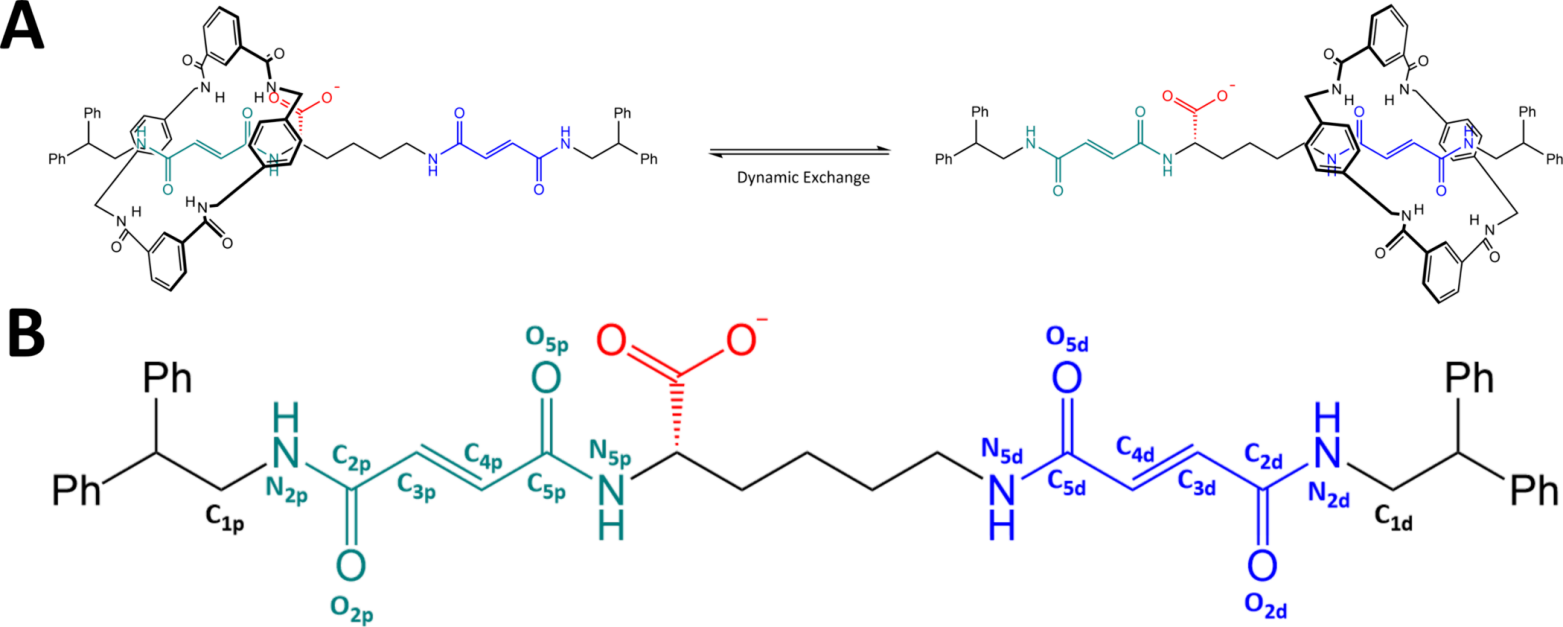
Molecular structure and dynamic exchange mechanism of the [2]­rotaxane system. (A) Schematic representation of the mechanism of shuttling in [2]­rotaxane: the macrocycle incessantly shuttles between two fumaramide binding sites (proximal station in turquoise, distal station in blue), spaced nonequidistantly from either side of a carboxylate moiety (red) on the axle. (B) Labels adopted in this paper for the axle’s atoms.

The carboxylate group catalyzes the fuel-to-waste reactions necessary for the activity of the information ratchet. In the first step, it acts as a nucleophile toward the carbodiimide fuel, generating a covalent intermediate (anhydride or ester) that can transiently hinder macrocycle motion. In the subsequent step, hydrolysis of the intermediate restores the original carboxylate, completing what functionally corresponds to a fuel-to-waste step. This cyclic conversion creates a kinetic asymmetry between the two stations: ester formation is favored when the macrocycle is at the distal station, while hydrolysis is enhanced when the macrocycle resides at the proximal one. Such asymmetry is consistent with the Curtin–Hammett principle, where the product distribution is governed by the relative energies of competing transition states rather than by the stability of ground-state conformers. Experimentally, additives like hydroxybenzotriazole (HOBt) or 1-hydroxy-7-azabenzotriazole (HOAt) have been employed to trap key intermediates and isolate conformational states within this cycle. In this way, the ratchet effect is powered by the chemical energy harvested from the fuel-to-waste reaction, while the molecular machine’s directionality is maintained by exploiting the differences in the reaction rates at the two stations, ensuring efficient and unidirectional operation.

Examining the initial equilibrium state of the rotaxane in the absence of fuel can provide key insights into the system’s inherent stability and behavior before the introduction of external energy. This state allows us to observe the macrocycle’s conformational preferences and the intramolecular interactions, such as hydrogen bonding between the amide moieties of the macrocycle and the fumaramide groups of the axle, which contribute to its stabilization at each station. By understanding these interactions, we can better characterize the thermodynamic properties of the system. Additionally, analyzing the equilibrium configuration allows us to chart the energy landscape and detect any intrinsic kinetic barriers that may influence the movement of the macrocycle. This knowledge is vital for understanding how the introduction of fuel changes the system’s dynamics, especially in terms of promoting unidirectional motion between the proximal and distal stations. Furthermore, this study sheds light on the passive role of the carboxylate group in maintaining stability and dictating the macrocycle’s distribution between the binding sites. To accurately capture the solvent’s potential role in modulating these intrinsic properties, we hypothesized that the distinct hydrogen-bond donor/acceptor capability of different solvents (in addition to their polarity) could significantly influence the rotaxane’s free-energy landscape. Such characteristic is commonly described by the empirical α (hydrogen-bond donating ability) and β (hydrogen-bond accepting ability) parameters.^66–71^ Although absolute values for these parameters vary across literature sources due to differences in experimental conditions and molecular probes, a general trend is consistently observed: chloroform (CHCl_3_) exhibits moderate hydrogen-bond donating ability and low polarity; dimethyl sulfoxide (DMSO) and acetonitrile (ACN) are both polar solvents, with DMSO being a stronger hydrogen-bond acceptor than ACN. Specifically, solvents like ACN and DMSO might stabilize intermediate rotaxane conformations primarily through ion–dipole interactions^72–75^ with the negatively charged carboxylate group, as well as through hydrogen bonding interactions with the amine groups of the fumaramide stations. Given their ability to act as hydrogen bond acceptors, these solvents would not be expected to compete directly with the macrocycle for key carbonyl acceptor sites, but rather stabilize specific states indirectly. Furthermore, ACN and DMSO are particularly useful solvents to test computationally, as they have been employed experimentally^35,36^ to evaluate the macrocycle distribution in the absence of fuel, providing not only valuable mechanistic insights into the system but also a direct means of validating our computational models.

Conversely, a solvent like CHCl_3_, despite its significantly lower polarity, might provide an alternative mode of stabilization due to its ability to donate hydrogen bonds via its polarized hydrogen atom.^76–80^ Unlike ACN and DMSO, CHCl_3_ could directly compete with the macrocycle for binding at the fumaramide carbonyl groups. Although expected to be relatively weak, such solvent–carbonyl hydrogen-bonding interactions would potentially alter the energetic landscape by occupying acceptor sites temporarily left exposed by the macrocycle during shuttling, thereby reducing energetic barriers and facilitating transitions between stable binding stations.

By analyzing these distinct solvent-mediated mechanisms through a combination of WT-MetaD and infrequent WT-MetaD simulations, we have comprehensively characterized the energetic landscape governing the rotaxane’s conformational states across different solvent conditions and provided quantitative kinetic information, including detailed transition times between the proximal and distal binding stations. Together, these simulations offer a molecular-level understanding of how solvent composition affects both the stability and dynamic behavior of the rotaxane. This detailed mechanistic insight lays the foundation for optimizing future designs and enhancing the efficiency of chemically fueled molecular machines.

## 2 Results and Discussion

All computational details, including simulation setup and the numerical methods, are provided in the Supporting Information.

### 2.1 Free-Energy Landscape and Solvent Effects on the Axle’s Flexibility

Unbiased molecular dynamics simulations were first performed in different solvents (ACN, DMSO, CHCl_3_, and water). Solubility was inferred by monitoring the radius of gyration: in water the system displayed a low value, consistent with a closed conformation, whereas in ACN, DMSO, and CHCl_3_ higher values indicated an extended conformation (Figure S1) in according with experimental data. Therefore, subsequent analyses focused on the first three solvents.

Moreover, the initial unbiased MD simulations revealed the complexity of rotaxane movement. At least three possible motions were found to be coupled together: the translational movement of the macrocycle on the axis, the conformational change of the latter and that of the axis itself, mainly due to the numerous degrees of freedom associated with the pentane linker between the fumaramide stations. Given the complexity of the ratchet and the limitations of unbiased MD simulations in sampling the system’s phase space at room temperature, WT-MetaD-based free energy calculations were employed. In WT-MetaD, standard MD is modified by introducing a history-dependent bias potential properly designed to drive the system in the exploration of the most relevant equilibrium conformations (and transitions) along one or more critical reaction coordinates represented by collective variables (CVs).

We selected two key collective variables to represent the reaction coordinate: the distances d and *d*_*CC*_. The latter measures the distance between *C*_*1p*_ and *C*_*1d*_ atoms, while the former uses the former value to calculate the relative position of the ring as a function of axis extension, according to the following formula:

$$d=\frac{p_{d}\times 2.7}{d_{cc}}$$

where *d* measures the distance between the center of mass (COM) of the 4 nitrogen atoms of the macrocycle and the carbon *C*_1*p*_ (Figure 2A, short yellow line); *p*_*d*_ then measures the projection of distance *d* along the *d*_*cc*_ axis (Figure 2A, short black line). The value of 2.7 nm represents the maximum achievable distance *d*_*CC*_ (Figure 2A, long black line). The conformational properties of the ring during the simulations were assessed a posteriori by calculating the dihedral angle ϕ, already effectively used as CV in the work of Fu et al.^59^ to discriminate the most relevant macrocycle configurations (Figure 2A, right).

**Figure 2:**
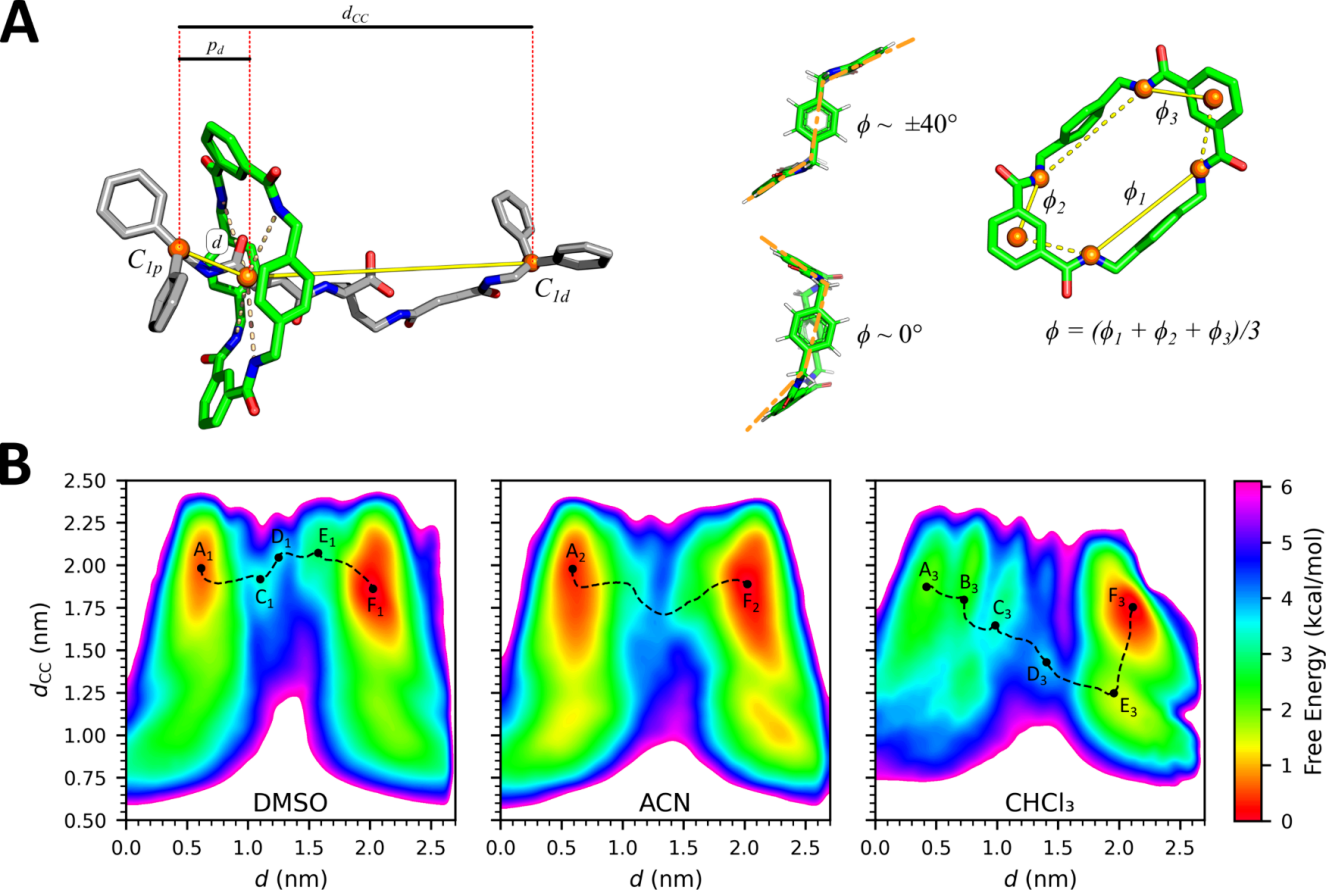
Collective variables and free-energy landscapes of the shuttling process in different solvents. (A) Schematic description of the [2]­rotaxane’s relevant movements. Left panel: structure of the [2]­rotaxane and the CVs (*d*, *d*_*CC*_) chosen to define the shuttling dynamics. Right panel: definition of the average dihedral angle ϕ, already used in Fu et al. work^59^ to describe the conformational change of the ring-like molecule, where ϕ = 0 denotes a boat-like conformation, while ϕ ≥ 20° or ϕ ≤ −20° indicates a chairlike conformation. (B) Two-dimensional free-energy surfaces for the shuttling process in DMSO, ACN, and CHCl_3_, as a function of the CVs *d* and *d*_*CC*_, obtained from well-tempered metadynamics simulations. The labels are assigned according to the following criterion: the capital letter indicates a common stage in the progression of the ring from one station to another (A is minima in the proximal station, F is minima in the distal station, B to E are metastable intermediates); the subscript refers to the solvent (1, 2, and 3 correspond to DMSO, ACN, and CHCl_3_, respectively).The black dashed lines represent the minimum free-energy paths connecting the main binding sites, as determined using the NEB algorithm. Color shading indicates the free energy in kcal/mol, as shown by the right-hand color bar.

Figure 2B shows the free-energy surface (FES) of the shuttling process, represented as a function of two collective variables, *d* and *d*_*CC*_. The WT-MetaD simulations unequivocally reveal that, across all tested solvents, the most stable conformations of the rotaxane correspond to states where the macrocycle is located close to the lateral fumaramide binding stations, while the shuttling occurs through less favored intermediate states.

For all solvents considered, the most stable structures feature the axle in a relatively extended conformation at both stations (1.76 nm < *d*_*CC*_ < 2.08 nm), stabilized by NH–O hydrogen bonds between the amide nitrogen atoms of the macrocycle and the carbonyl groups of the axle. A preferential interaction is observed with the carbonyl groups belonging to the carboxylate moiety on the distal station: structural analyses show that the pentane linker adopts a conformation allowing the carboxylate group to point directly toward the center of the macrocycle (aligned with the axis of the distal station), thereby maximizing favorable interactions both with the ring and with the distal station itself. The shuttling process thus requires the ring to ″walk″ along the axle by sequentially breaking and reforming hydrogen bonds, not only within the rotaxane but also involving the surrounding solvent molecules. Interestingly, once the ring leaves the fumaramide stations, the axle exhibits an increased tendency to bend in less polar solvents, maximizing the number of hydrogen bonds formed between the axle and the macrocycle.

### 2.2 Solvent-Dependent Effects on the Shuttling Mechanism

To explore the solvent impact more thoroughly and to gain further insight into the factors governing the equilibrium behavior of the rotaxane, we decomposed the global free-energy profiles (ΔG) into their enthalpic (ΔH) and entropic (−TΔS) contributions. This decomposition was performed along the minimum free-energy path connecting the two most stable minima, corresponding to the macrocycle bound at the fumaramide stations. The path was computed using the nudged elastic band (NEB) algorithm^81,82^ applied to the two-dimensional FES obtained for each solvent. This approach allowed us to isolate the most probable shuttling route and perform all subsequent analyses along a physically meaningful transition coordinate, such as enthalpy, entropy, structural properties, and interactions. We employed a validated framework for the energy decomposition,^64^ recently shown to be effective in resolving early stage nucleation events in metal–organic frameworks^65^ and in analyzing solvent-modulated dynamics in rotaxane systems.^63^

The first solvent considered is DMSO, a polar solvent with the ability to accept hydrogen bonds. The free-energy profile computed for the rotaxane in DMSO (Figure 3A) also reveals two main minima near the recognition sites, located at *d* ∼0.6 nm and *d* ∼2.0 nm from *C*_*1p*_, with the axle adopting a relatively extended conformation (*d*_*CC*_ ∼1.9 nm). Once again, the free-energy difference between the two minima is small (ΔG_F1‑A1_ = -0.55 ± 0.2 kcal/mol), in agreement with experimental observations. However, at least three metastable states were detected between the two binding sites (labeled C_1_, D_1_, and E_1_). At C_1_ (*d* ∼1.1 nm, *d*_*CC*_ ∼1.9 nm, ΔG_F1–C1_ = -3.36 kcal/mol), the macrocycle partially detaches from the axle and binds to the carboxylate group near the proximal station (Figure 3B), forming approximately one additional hydrogen bond compared to the proximal binding state. In this configuration, the carboxylate also interacts with the distal fumaramide unit (∼0.5 H-bond). Nonetheless, the instability of this intermediate arises primarily from the displacement of solvent molecules: a DMSO molecule previously interacting with the proximal fumaramide and another with the carboxylate (via ion–dipole interactions) are expelled, and the entropic stabilization gained does not fully compensate for the enthalpic loss. At D_1_ (*d* ∼1.25 nm, *d*_*CC*_ ∼2.0 nm, ΔG_F1‑D1_ = -3.53 kcal/mol), the ring remains partially bound to the proximal fumaramide but simultaneously interacts with the carboxylate, now oriented toward the distal station and partially engaging with the hydrophobic linker. This rearrangement leads to a reduction in the number of H-bonds between the carboxylate and the ring, but allows DMSO molecules to reestablish interactions with the proximal fumaramide, partially stabilizing this configuration. At E_1_ (*d* ∼1.6x nm, *d*_*CC*_ ∼2.1 nm, ΔG_F1‑E1_ = −2.91 kcal/mol), the macrocycle predominantly wraps around the hydrophobic linker, maintaining partial interactions with both the carboxylate and a distal carbonyl group.

**Figure 3:**
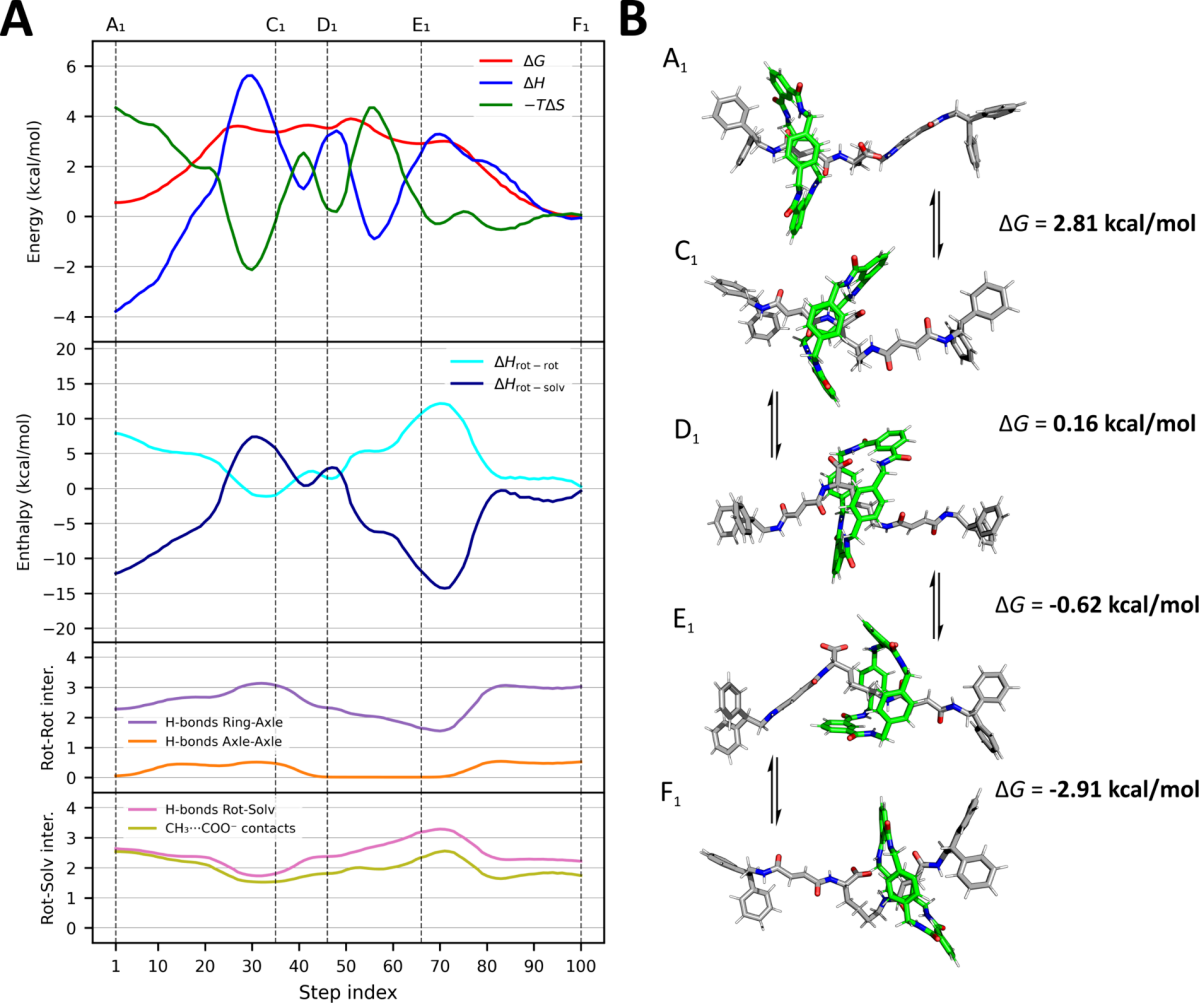
Thermodynamic and structural characterization of the shuttling process in DMSO. (A) Energetic and interaction profiles along the minimum free-energy pathway connecting the two primary binding sites (A_1_ and F_1_), as identified by the NEB algorithm using 100 discrete steps and projected onto a two-dimensional collective variable space. The *x*-axis (step index) represents trajectories of 100 frames, illustrating the translocation of the ring from the proximal to the distal station in the three different solvents. The trajectories were obtained by selecting the 100 frames that best correspond to the paths reported in Figure 2B (the dashed black lines).The top plot shows the decomposition of the free-energy profile (Δ*G*, red) into its enthalpic (Δ*H*, blue) and entropic (−*T*Δ*S*, green) contributions along the path. Vertical dashed lines mark the position of all free-energy minima along the pathway, including the two main binding sites (A_1_ and F_1_) and the metastable intermediates (C_1_, D_1_, E_1_), as identified from local minima in the ΔG profile. The middle plot separates the total enthalpy (ΔH) into intrarotaxane (Δ*H*_rot‑rot_, cyan) and rotaxane–solvent (Δ*H*_rot‑solv_, dark blue) contributions, highlighting the distinct energetic roles of solute–solute and solute–solvent interactions. The two bottom plots report interaction counts along the pathway: the third displays intrarotaxane contacts, including hydrogen bonds between the macrocycle and the axle (purple) and between different regions of the axle (orange); the fourth panel shows solvent-mediated interactions, including hydrogen bonds between the rotaxane and DMSO (pink) and CH_3_···COO^–^ ion–dipole contacts involving DMSO methyl groups (olive). (B) Representative conformations of the rotaxane along the shuttling path, corresponding to the main metastable states (A_1_, C_1_, D_1_, E_1_, F_1_). The macrocycle and the axle are shown in green and gray, respectively. Arrows indicate transitions between states, and the associated Δ*G* values (in kcal/mol) represent the free-energy difference between each configuration and the preceding one along the pathway (e.g., Δ*G* between A_1_ and C_1_ is calculated as G­(C_1_) – G­(A_1_)). The sequence of conformational snapshots illustrates the progressive detachment of the macrocycle from the proximal station (A_1_), its partial engagement with the carboxylate group and hydrophobic linker (C_1_–E_1_), and its final stabilization at the distal station (F_1_).

The energy decomposition (Figure 3A, first plot) shows that the enthalpy profile between the two binding stations again features four minima, two of which correspond to the primary binding sites of the rotaxane. At these positions, the macrocycle establishes hydrogen bonds with both the fumaramide carbonyl groups and the carboxylate group (∼2.3 H-bonds for the proximal station and ∼3 H-bonds for the distal station). Furthermore, when the ring is located on the distal station, the carboxylate additionally interacts with the distal fumaramide (∼0.5 H-bond), thanks to the flexibility of the pentane linker, maximizing favorable solute–solute interactions. Interestingly, although the distal station exhibits a slightly greater number of total hydrogen bonds, the proximal station is enthalpically more stable by approximately 4 kcal/mol.

The decomposition of ΔH into intrarotaxane ΔH_rot‑rot_ and rotaxane–solvent ΔH_rot–solv_ contributions (Figure 3A, second plot) shows that the proximal station benefits from a greater number of rotaxane–solvent interactions: hydrogen bonds between the axle and solvent are slightly more numerous when the macrocycle is positioned at the proximal station (∼2.5 H-bonds) compared to the distal station (∼2 H-bonds). Additionally, a higher number of ion–dipole interactions between the methyl hydrogens of DMSO and the negatively charged carboxylate (∼2.5 contacts for the proximal station vs less than 2 for the distal station), further contributes to this stabilization. Because of these enhanced solvent–rotaxane interactions, the entropic contribution disfavors the proximal station compared to the distal one. Regarding the two intermediate enthalpic minima (*d* ∼1.2 nm and *d* ∼1.5 nm), their energy is comparable to that of the distal binding site. The first corresponds to a configuration where the macrocycle is partially bound to a proximal carbonyl and positioned directly above the carboxylate, in a transition state between minima C_1_ and D_1_. The second intermediate corresponds to the macrocycle located around the hydrophobic linker, partially interacting with both the carboxylate and a distal carbonyl group. Interaction analyses (Figure 3A, third and fourth plots) and ΔH decomposition reveal that, these two intermediates are stabilized by different mechanisms: In the first case, the enthalpic contributions from intrarotaxane and rotaxane–solvent interactions are balanced, while in the second, stabilization is dominated by solvent–rotaxane interactions. The high polarity and strong negative partial charge on DMSO oxygen atoms promote frequent binding to the rotaxane during shuttling. This is accompanied by entropic maxima at these points, explaining why they do not correspond to metastable states on the shuttling path.

On the entropic side, we observed at least four distinct minima at *d* ∼1.0 nm, *d* ∼1.25 nm, *d* ∼1.7 nm, and *d* ∼1.85 nm. The first minimum, which is also the deepest, corresponds to a state characterized by the lowest number of interactions between the rotaxane and the solvent, significantly reducing configurational freedom. The other three entropic minima are energetically comparable but differ notably in their solvation patterns. The second (*d* ∼1.25 nm) and the fourth (*d* ∼1.85 nm) minima both exhibit reduced carboxylate solvation, primarily through interactions with the methyl groups of DMSO. Conversely, the third minimum at *d* ∼1.7 nm features maximal rotaxane–solvent interactions, with extensive hydrogen bonds and ion–dipole contacts. In this case, the entropy minimum arises predominantly from increased conformational freedom of the rotaxane, as the macrocycle resides on the pentane linker and experiences fewer restrictive interactions with the axle. This subtle interplay of solvation and conformational mobility significantly shapes the rotaxane’s overall entropic landscape. To sum up, in DMSO the macrocycle remains almost equally distributed between the two stations, yet the energetic contributions are asymmetric: enthalpy favors the proximal state due to stronger rotaxane–solvent interactions, while entropy promotes the distal occupation.

Another solvent examined is acetonitrile (ACN), which exhibits lower polarity than DMSO and a reduced propensity for hydrogen-bond acceptance. In ACN, the projection of the free-energy surface onto the *d* variable (Figure 4A) reveals two main minima located near the binding stations, at approximately *d* ∼0.6 nm and *d* ∼2.0 nm from *C*_*1p*_, with the axle adopting a relatively extended conformation (*d*_*CC*_ ∼1.9 nm). The free-energy difference between the two minima is small (ΔG_F2‑A2_ = -0.3 ± 0.4 kcal/mol), indicating that the two conformations are almost isoenergetic, in agreement with the experimentally observed distribution of the macrocycle.^35,36^ No significant metastable states were detected between the two binding stations, unlike the rotaxane’s behavior in DMSO: the weaker solvent–rotaxane interactions in ACN (through hydrogen bonds and ion–dipole interactions) allow for relatively less stable configurations when the macrocycle leaves the binding stations.

**Figure 4:**
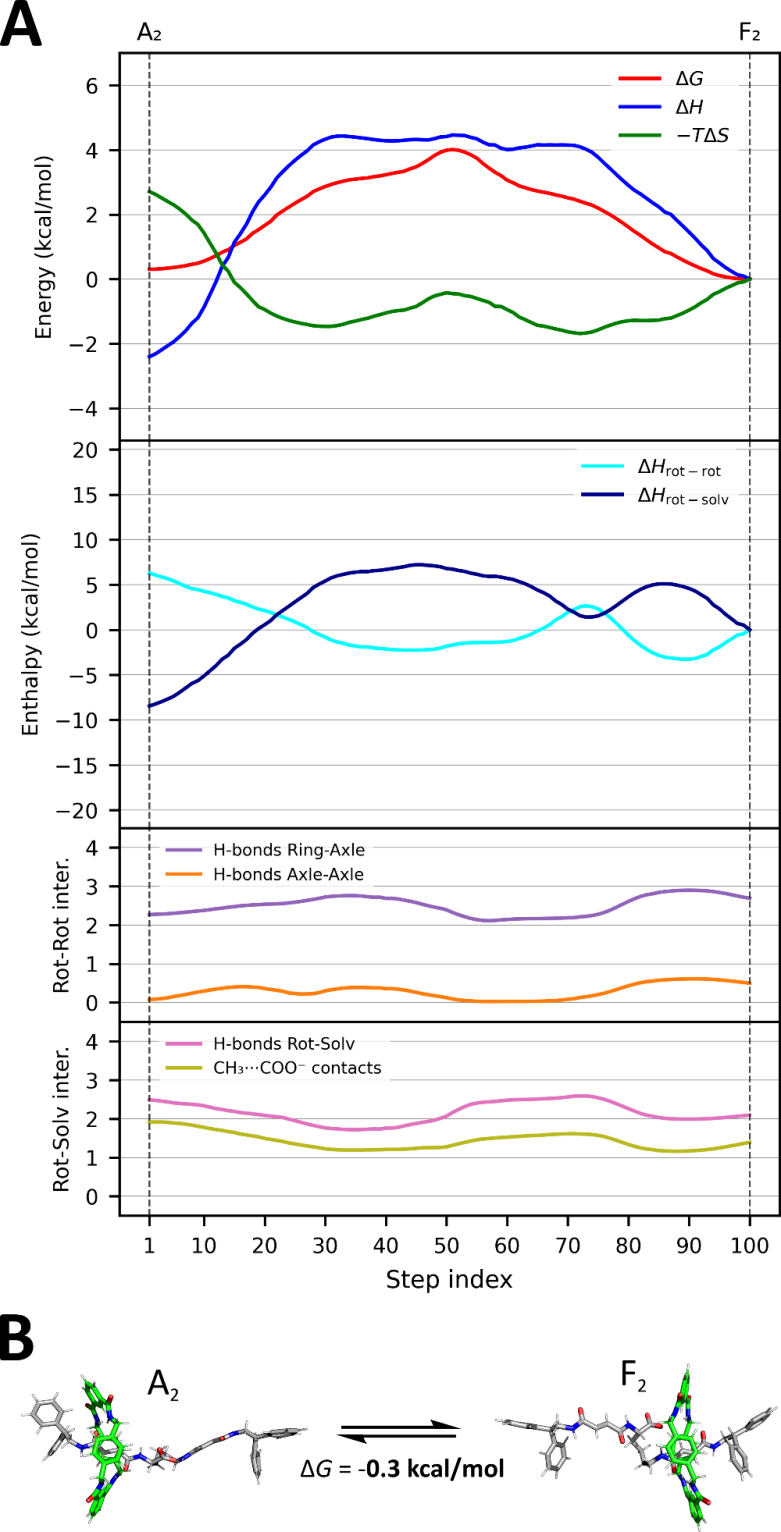
Thermodynamic and structural features of the shuttling process in ACN. (A) Energetic and interaction profiles along the minimum free-energy path connecting the two primary binding sites (A_2_ and F_2_), as identified using the NEB algorithm and projected onto a two-dimensional collective variable space. The *x*-axis (step index) represents trajectories of 100 frames, illustrating the translocation of the ring from the proximal to the distal station in the three different solvents. The trajectories were obtained by selecting the 100 frames that best correspond to the paths reported in Figure 2B (the dashed black lines).The top panel shows the decomposition of the total free energy (Δ*G*, red) into its enthalpic (Δ*H*, blue) and entropic (−*T*Δ*S*, green) components. Vertical dashed lines indicate the position of the two main binding states. The middle panel reports the decomposition of Δ*H* into intrarotaxane (Δ*H*_rot–rot_, cyan) and rotaxane–solvent (Δ*H*_rot–solv_, dark blue) contributions. The bottom two panels quantify interaction counts along the path: intrarotaxane interactions include hydrogen bonds between the macrocycle and the axle (purple) and between different parts of the axle itself (orange); rotaxane–solvent interactions are shown as hydrogen bonds with ACN molecules (pink) and CH_3_···COO^–^ ion–dipole contacts (olive). (B) Representative structures of the rotaxane at the two main free-energy minima (A_2_ and F_2_), highlighting the extended conformations stabilized by ring–station hydrogen bonding, where no clear metastable intermediates are observed along the path.

The free energy profile (global ΔG, red line) was decomposed into enthalpic (ΔH, blue line) and entropic (−TΔS, green line) contributions (Figure 4A, first plot). The enthalpic profile exhibits four minima in total, two of which correspond to the main recognition stations: here the macrocycle establishes multiple hydrogen bonds with the axle, interacting with both the carbonyl groups of the fumaramide units and the carboxylate group (∼2.3 H-bonds for the A2 and ∼2.8 for F_2_). Additionally, when the ring is located at the distal station, the carboxylate group tends to form more extensive interactions with the distal fumaramide group due to the flexibility of the pentane linker, further maximizing favorable solute–solute interactions (Figure 4B). Interestingly, although the distal station exhibits a slightly greater number of total hydrogen bonds, the proximal station is enthalpically more stable by approximately 2.5 kcal/mol.

The further decomposition of ΔH into the contributions from intrarotaxane interactions (ΔH_rot‑rot_) and rotaxane–solvent interactions (ΔH_rot–solv_) reveals that the proximal station is additionally stabilized by a slightly higher number of solvent-mediated interactions (Figure 4A, second plot). Moreover, a greater number of ion–dipole interactions between the methyl hydrogens of ACN and the carboxylate group (∼2 contacts for the proximal vs ∼1.5 for the distal station) further stabilizes the proximal configuration (Figure 4A, third and fourth plots). Because of this enhanced solvent interaction, the entropic contribution disfavors the proximal station compared to the distal one. This observation is consistent with the high polarity of ACN, although its weaker hydrogen-bond acceptor capabilities respect to DMSO, which result in a less pronounced stabilization of the rotaxane–solvent interactions. Regarding the two intermediate enthalpic minima (located at *d* ∼1.1 nm and *d* ∼1.6 nm), they are less stable than the main binding sites by ∼4 kcal/mol and correspond to configurations where the macrocycle is positioned close to the carboxylate.

Interaction analyses and decomposition of ΔH, show that these intermediates are primarily stabilized through direct macrocycle–axle interactions. Specifically, at *d* ∼1.1 nm, the macrocycle forms its maximum number of hydrogen bonds with the axle (∼3 H-bonds), whereas at *d* ∼1.6 nm, only two hydrogen bonds are formed. Thus, ΔH_rot‑rot_ is higher at *d* ∼1.6 nm, partially compensating for the energy gap through additional hydrophobic interactions between the linker and the benzyl groups of the macrocycle. Moreover, at this position, the rotaxane forms approximately the same H-bonds with the solvent as when the macrocycle is on the proximal station, which also contributes to partially stabilizing the configuration and mitigating the overall energy penalty associated with reduced axle–macrocycle interactions.

On the entropic side, two minima are observed, coinciding with the enthalpic maxima at *d* ∼1.0 nm and *d* ∼1.7 nm: respect to the minimum at the proximal station, the first entropic minimum results from the loss of ∼0.5 H-bonds and ∼1 ion–dipole interaction between the axle and solvent, while the second arises from the loss of approximately 1 H-bond between the axle and the ring and ∼0.5 H-bond between the carboxylate and the distal fumaramide station, increasing the mobility of the rotaxane in a way similar to what happens in DMSO. Overall, the macrocycle distributes itself approximately equally between the two stations, with individual energetic contributions still asymmetric like DMSO.

The last solvent considered is chloroform (CHCl_3_), which, unlike the previous ones, is nonpolar and can act only as a hydrogen-bond donor. The dynamics of the macrocycle along the axle in chloroform differ significantly from those observed in the other two solvents. This behavior reflects the distinct physicochemical properties of CHCl_3_ compared to DMSO and ACN: chloroform is weakly polar and a hydrogen-bond donor, while DMSO and ACN are highly polar and primarily hydrogen-bond acceptors. Inspection of the FES and the corresponding free-energy profiles connecting the minima associated with the macrocycle at the fumaramide stations (*d* ∼0.45 nm and *d* ∼2.1 nm; Figure 5A, first plot) reveals that the low polarity of CHCl_3_ promotes shuttling through configurations where the rotaxane axle adopts a more flexible and collapsed structure, minimizing its exposure to the solvent (Figure 5B and S2). Furthermore, because CHCl_3_ can directly compete with the macrocycle for binding to the fumaramide carbonyl groups, the ring encounters less resistance during shuttling compared to DMSO and ACN, where the solvents form stabilizing hydrogen bonds with the axle that must be disrupted to allow ring passage. However, in CHCl_3_, the hydrogen bonds formed between the solvent and polar groups at the unoccupied metastable spot (most notably involving the carboxylate moiety, see Figure S3) must still be broken during the shuttling. These interactions, although individually weak, appear collectively significant and stabilizing, particularly in regions where the carboxylate is transiently exposed. The free-energy difference ΔG_F3‑A3_ between the two stations is −2.0 ± 0.6 kcal/mol, favoring the macrocycle at the distal station. Our data suggest that macrocycle distribution in absence of fuel is not necessarily symmetric across all solvents. Low-polarity environments can bias the macrocycle toward the distal station even under equilibrium conditions, questioning the assumption of initial 1:1 station occupation used in kinetic modeling, which holds only for DMSO and ACN solvents as reported in literature.^35,36^ This raises the possibility of a solvent-induced asymmetry affecting both the directionality and speed of autonomous operation.

**Figure 5:**
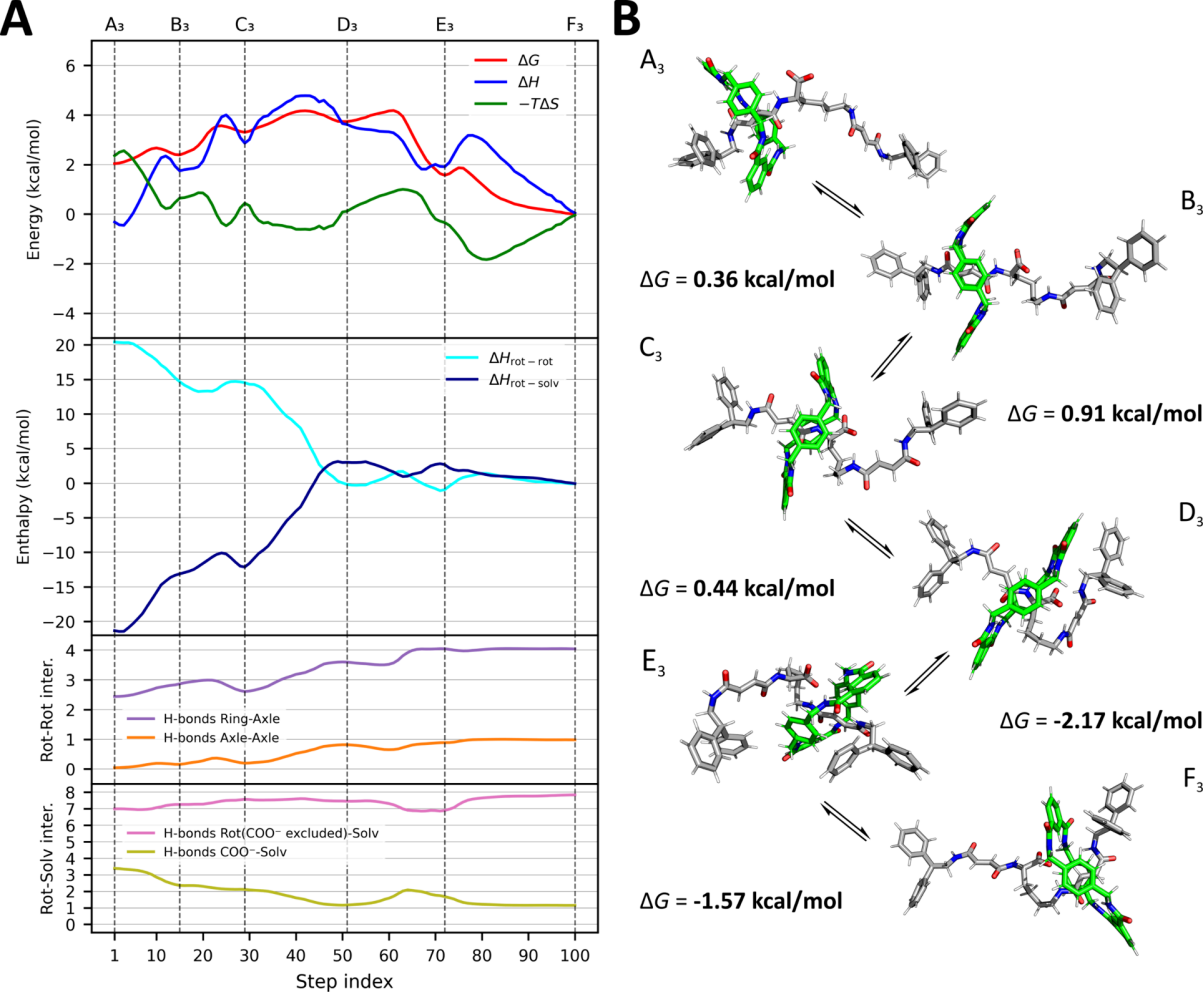
Thermodynamic and structural characterization of the shuttling process in CHCl_3_. (A) Energetic and interaction profiles along the minimum free-energy pathway connecting the two primary binding sites (A_3_ and F_3_), as identified by the NEB algorithm using 100 discrete steps and projected onto a two-dimensional collective variable space. The *x*-axis (step index) represents trajectories of 100 frames, illustrating the translocation of the ring from the proximal to the distal station in the three different solvents. The trajectories were obtained by selecting the 100 frames that best correspond to the paths reported in Figure 2B (the dashed black lines). The top plot shows the total free energy (Δ*G*, red) decomposed into enthalpic (Δ*H*, blue) and entropic (−*T*Δ*S*, green) components. Vertical dashed lines mark the positions of all local minima along the path, including metastable intermediates (B_3_, C_3_, D_3_, E_3_) and the binding sites (A_3_, F_3_), based on local minima in the Δ*G* profile. The middle plot shows the decomposition of ΔH into intrarotaxane (Δ*H*_rot–rot_, cyan) and rotaxane–solvent (Δ*H*_rot–solv_, dark blue) contributions, emphasizing the evolving roles of internal and solvent-mediated interactions. The two bottom plots report interaction counts along the pathway: the upper one displays intrarotaxane contacts, including hydrogen bonds between the macrocycle and the axle (purple) and between different regions of the axle (orange); the lower one reports rotaxane-solvent interactions, shown as hydrogen bonds between rotaxane and solvent (carboxylate excluded, pink) and CHCl_3_···COO^–^ hydrogen bonds (olive). (B) Representative conformations sampled along the shuttling pathway, corresponding to the most relevant local minima. The macrocycle and axle are shown in green and gray, respectively. Transitions between states are indicated with arrows, and the associated Δ*G* values (in kcal/mol) represent the relative free-energy differences between each minimum and its predecessor along the path. The trajectory captures the progressive rearrangement of the macrocycle as it moves away from the proximal binding site (A_3_), sequentially engages with the carboxylate group and linker (B_3_–E_3_), and ultimately reaches the distal station (F_3_), where favorable intrarotaxane interactions are maximized.

While the enthalpic contributions to both stations are similar, the proximal station is entropically penalized, reflecting the stronger solvation of the carboxylate in this position. The metastable states observed along the shuttling pathway (B_3_, C_3_, D_3_, and E_3_) correspond to progressively collapsed conformations of the axle, favoring desolvation of the carboxylate and maximizing direct interactions between the axle and the macrocycle. In this solvent, weak but direct hydrogen bonds involving the solvent’s polarized hydrogen atom promote the transient stabilization of metastable conformations. These intermediate states are energetically comparable to the proximal binding station (unlike in the other solvents) and are stabilized through a unique balance of enthalpic interactions and entropic penalties. Throughout this process, the ring gradually moves toward the distal station, and up to D_3_, the transitions are entropically favored. At B_3_ (*d* ∼0.7 nm), the carboxylate begins to approach the distal fumaramide while simultaneously losing approximately one hydrogen bond with the solvent. This is followed by state C_3_ (*d* ∼1.0 nm), where the ring starts to interact partially with the carboxylate, further promoting its desolvation. In state D_3_ (*d* ∼1.4 nm), both amide groups of the fumaramide units (after rotation of the *C*_*2d*_–*C*_*3d*_ bond, see Figure 1B for labels) begin to interact with the now largely desolvated carboxylate. At this point, the number of chloroform molecules hydrogen-bonded to the charged carbonyls drops significantly. Finally, in E_3_ (*d* ∼1.9 nm), the macrocycle completes the crossing toward the distal side of the axle, which remains in a highly folded conformation (Figure 5B). The transition from E_3_ to the final distal minimum F_3_ involves the extension of the axle and the maximization of intrarotaxane hydrogen bonding (∼5 H-bonds), inversely correlated with a decrease in rotaxane–solvent interactions. The enthalpic minima along the pathway correspond to those observed on the energy path, except for D_3_: here, the enthalpy profile shows a negative derivative, driving the system toward E_3_, where rotaxane–rotaxane contacts are maximized. This transition passes through a less favorable entropic region where the carboxylate is more solvated compared to D_3_.

Further decomposition of ΔH into intrarotaxane ΔH_rot‑rot_ and rotaxane–solvent ΔH_rot–solv_ contributions (Figure 5A, second plot) reveals that, as in other solvents, the proximal station is stabilized by a higher number of rotaxane–solvent interactions: specifically, the number of hydrogen bonds between the carboxylate and solvent is greater when the macrocycle is at the proximal station (∼3.5 H-bonds) compared to the distal station (∼1 H-bond). These strong interactions with the charged carbonyls compensate for the fewer ring–axle and carboxylate–axle hydrogen bonds (∼2.5 H-bonds at proximal vs ∼5 H-bonds at distal). This behavior arises because, despite its low polarity, chloroform can act as a weak hydrogen-bond donor via its single polarized hydrogen atom, directly competing with the macrocycle and stabilizing the rotaxane configuration when the ring leaves the proximal fumaramide station. These findings carry direct implications for the autonomous operation of the fuel-driven ratchet. The authors of the original experimental studies^35,36^ postulate that steric hindrance in the proximal state slows down fuel attachment, while hydrogen bonding in the same state accelerates hydrolysis of the barrier-forming species. Our analysis suggests that solvent-mediated stabilization of intermediates and transition states may be equally important in determining the efficiency of the ratchet mechanism.

Based on our findings, we propose an alternative mechanism for fuel-to-waste catalysis. Although our simulations suggest that the distal station places the carboxylate group in a configuration that appears sterically shielded and thus less directly accessible in all the tested solvents, the experimentally observed increased reactivity for the fuel-to-waste step at the distal site could instead result from subtle yet significant structural preorganization. Specifically, although partial shielding of the carboxylate reduces direct solvent accessibility, the interactions lost with the solvent are compensated by increased coordination with the distal fumaramide and the macrocycle itself. This intramolecular coordination positions the carboxylate in a structurally organized, prereactive conformation. Although partial coordination with the distal fumaramide and macrocycle may slightly decrease the intrinsic nucleophilicity of the carboxylate oxygens by partially occupying their electron pairs, this arrangement significantly reduces solvation and entropy-related barriers. As a result, the overall activation energy required to achieve the fuel-to-waste reaction pathway could be lowered due to a more favorable structural orientation and decreased energetic cost for conformational reorganization, rather than through direct enhancement of nucleophilicity.

### 2.3 Solvent modulation of the Δ*H* vs −*T*Δ*S* relationship

Analysis of the ΔH vs –TΔ*S* scatter plots highlights a clear difference in enthalpy–entropy compensation across the three solvents (Figure S4). In DMSO (r ≈ –0.82) and ACN (r ≈ –0.85), a strong negative correlation is observed, consistent with a regime of tight compensation. Mechanistically, whenever the rotaxane gains enthalpic stabilization through specific interactions, such as directional hydrogen bonds in DMSO or ACN, the system becomes more rigid, leading to a proportional entropic penalty. In these polar solvents, enthalpic and entropic contributions are therefore tightly coupled, preventing simultaneous gains in both. In contrast, in CHCl_3_ the correlation is much weaker (r ≈ –0.37), indicating a partial breakdown of enthalpy–entropy compensation. Because chloroform interacts only weakly and transiently with the rotaxane, it retains greater conformational freedom. As a result, it can access states where favorable enthalpy is not counterbalanced by a corresponding entropic penalty, and vice versa, where entropically favorable states are not associated with strong enthalpic losses. In this low-polarity environment, ΔH and –TΔ*S* are effectively “decoupled”, suggesting that shuttling barriers in apolar media are more strongly modulated by individual energetic contributions rather than by a rigid enthalpy–entropy balance.

### 2.4 Mechanistic Implications on Kinetic Modulation by the Solvent Environment

The estimation of free-energy barriers among all identified minima was performed using infrequent WT-MetaD (details provided in the Supporting Information). The shuttling dynamics generally involve intermediate steps between the two fumaramide stations, with the exception of the rotaxane in ACN: the energy barriers for the proximal-to-distal A_2_-F_2_ and distal-to-proximal F_2_-A_2_ transitions are relatively symmetric, with similar values (12.6 ± (0.3, 0.7) kcal/mol for proximal-to-distal and 13.5 ± (0.3, 0.7) kcal/mol for distal-to-proximal (Figure 6), consistent with the nearly isoenergetic nature of the two stations. In both directions, the transition state corresponds to a configuration where the ring simultaneously interacts with the carbonyl oxygen *O*_*5p*_ of the proximal fumaramide and the carboxylate group. In both cases, the maximum of the free-energy profile coincides with a minimum in the total number of hydrogen bonds along the pathway. The height of these barriers is compatible with a slow shuttling process, with transition times in the microsecond to millisecond range.

**Figure 6:**
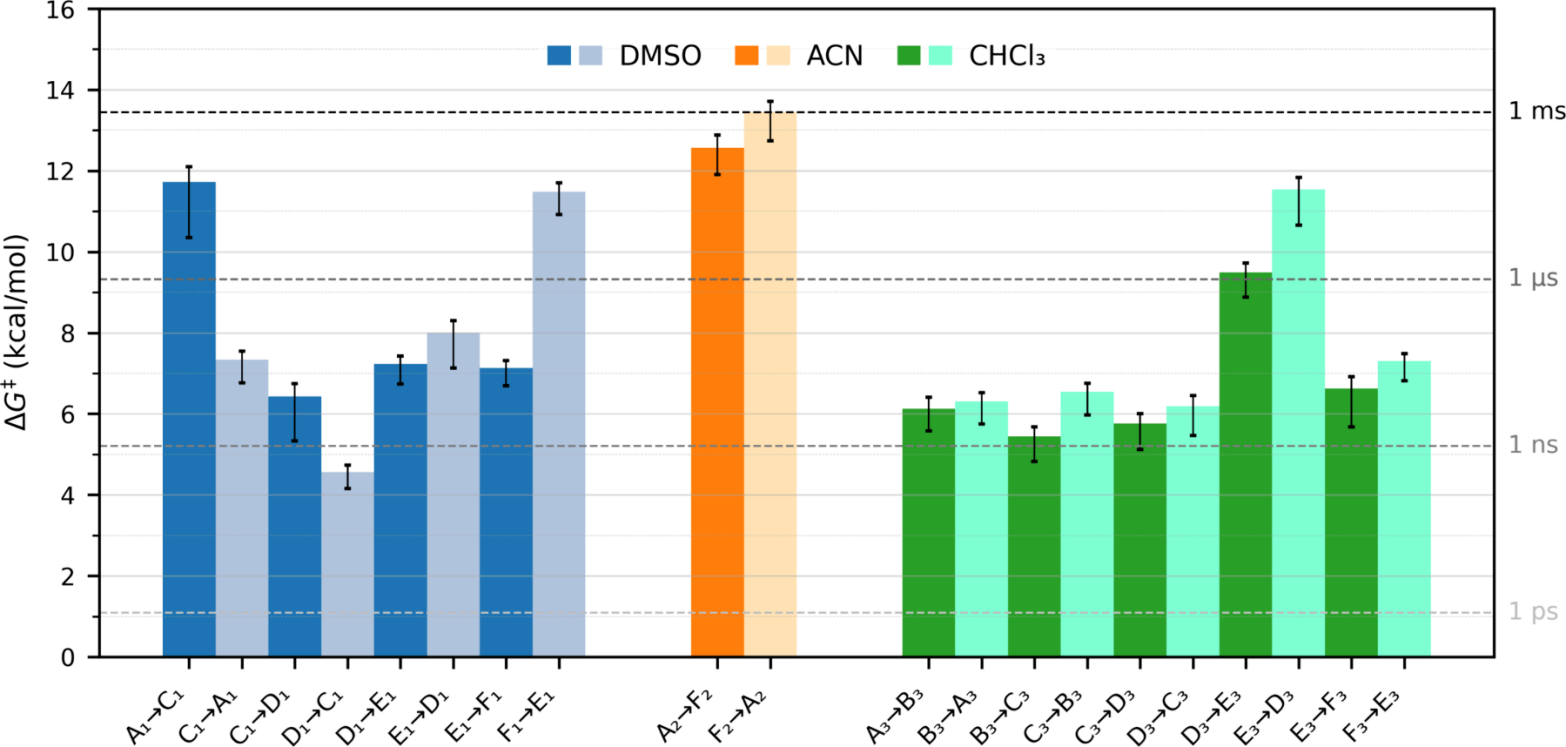
Free-energy barriers (Δ*G* ^‡^) for the shuttling transitions in different solvents. Bar chart reporting the activation free energies computed from infrequent WT-MetaD simulations for selected transitions along the minimum free-energy path in DMSO (blue), ACN (orange), and CHCl_3_ (green). Each bar represents a distinct transition between local minima identified from the WT-MetaD energy landscape along the shuttling path. Forward and reverse transitions are shown side-by-side for comparison. Darker bars indicate forward directions (proximal-to-distal), while lighter bars correspond to reverse transitions. Dashed horizontal lines mark the approximate Δ*G* ^‡^ values corresponding to typical transition time scales based on Eyring theory: 1 ps (∼1.1 kcal/mol), 1 ns (∼5.2 kcal/mol), 1 μs (∼9.3 kcal/mol), and 1 ms (∼13.5 kcal/mol) at 300 K. These time scale references allow for immediate visual comparison of kinetic barriers across solvent environments.

In contrast, the free-energy profiles obtained for the same rotaxane in DMSO and CHCl_3_ display different behavior, characterized by the presence of metastable intermediate states between the two fumaramide stations, as previously discussed (Figure 6). In DMSO, the two major barriers correspond to configurations where the macrocycle partially detaches from the axle, breaking hydrogen bonds with the station and disrupting ion–dipole contacts with the solvent. In both cases, these transitions occur slightly faster than in ACN (ΔG^‡^_A1–C1_ = 11.7 ± (0.4, 1.4) kcal/mol; ΔG^‡^_F1–E1_ = 11.5 ± (0.4, 1.4) kcal/mol), and subsequent rearrangements between intermediates are considerably faster (nano- to picosecond range), indicating that once rate-limiting barriers are crossed, shuttling proceeds efficiently.

In CHCl_3_, the shuttling pathway is more rugged, involving at least four intermediate states before the ring crosses the carboxylate moiety and moves to the distal station. Specifically, only after crossing from minimum D_3_ to E_3_ (ΔG^‡^_D3–E3_ = 9.5 ± (0.2, 0.6) kcal/mol) does the ring effectively engage with the distal fumaramide station. The transitions from minima B_3_, C_3_, and D_3_ involve lower barriers, associated with faster time scales (nanoseconds), as does the transition from E_3_ to the absolute minimum F_3_, where the axle extends from a folded to a stretched structure. When considering the reverse shuttling direction (distal to proximal), the highest barrier corresponds to the transition from E_3_ to D_3_ (ΔG^‡^_E3–D3_ = 11.5 ± (0.3, 0.9) kcal/mol), where the strong enthalpic stabilization of E_3_ renders the crossing kinetically slower compared to the forward direction. Our enthalpy–entropy decomposition reveals that CHCl_3_, as a hydrogen-bond donor, transiently compensates for the absence of the macrocycle at the fumaramide stations by interacting with available carbonyl groups. This solvent-mediated stabilization lowers the energetic cost of conformational rearrangements and facilitates smoother shuttling, especially in regions where the ring vacates hydrogen-bond acceptor sites. Thus, CHCl_3_ mimics aspects of catalytic transition-state stabilization.

Moreover, the steric bulk and spatial organization of the solvent shell appear to restrict ring mobility, especially in DMSO and CHCl_3_, where the confined environment contributes to barrier formation. Analysis of the macrocycle torsion angle ϕ (Figure S2) reinforces this: in ACN, fluctuations are minimal, whereas in DMSO and CHCl_3_, significant angular shifts suggest forced adaptation to solvent-imposed constraints.

Overall, the conformational free-energy landscape is strongly modulated by the solvent. In DMSO, barriers arise from both enthalpic and entropic penalties, particularly due to solvent structuring around exposed polar regions. In CHCl_3_, enthalpic stabilization dominates, and transitions occur through entropically favorable, disordered configurations. Conversely, ACN’s smoother profile reflects its linear geometry and moderate polarity, enabling easier solvent rearrangement. Despite these differences, the main rate-limiting steps remain in the microsecond regime across all systems. Nonetheless, the nature and symmetry of the barriers vary subtly with solvent identity, highlighting the delicate balance between enthalpic driving forces, solvation effects, and conformational flexibility in controlling rotaxane dynamics.

These findings also carry implications for understanding and optimizing autonomous, fuel-driven operation in experimental systems. Although pure water was excluded from our simulations due to precipitation issues observed both computationally and experimentally, Borsley et al. employed mixed ACN/H_2_O (ratio 70:30) solvents^35,36^ to facilitate hydrolytic barrier removal during the fuel-to-waste reaction. In these systems, water acts not only as a nucleophile but may also contribute to kinetic gating by modulating the electrophilicity of the activated intermediates (this aspect will be investigated through quantum mechanical calculations).

While this gating effect has been primarily attributed to macrocycle-mediated polarization of the activated ester, our data suggest a complementary or alternative explanation. Specifically, the greater solvent accessibility of the carboxylate group when the ring occupies the proximal station could enable water molecules to directly stabilize and polarize reactive intermediates, enhancing their electrophilicity through hydrogen-bonding and dielectric effects. Such solvent-driven stabilization may play a key role in accelerating hydrolysis of the barrier-forming species. Future QM simulations incorporating explicit water molecules and mixed-solvent models could provide deeper insight into this behavior, clarifying the molecular determinants of catalytic efficiency and kinetic control in dissipative molecular machines.

## 3 Conclusions

Rotaxanes have emerged as pivotal components in the design of artificial molecular machines, offering unique topological features and mechanically interlocked architectures that enable controlled motion along molecular tracks. Among these, [2]­rotaxanes capable of fuel-driven directional shuttling have attracted particular attention as minimal models of chemically powered molecular motors. Understanding how environmental factors like solvent polarity and molecular flexibility influence the conformational energy landscape is essential for uncovering the principles behind their autonomous behavior and catalytic efficiency.

In this work, we combined enhanced sampling techniques and thermodynamic decomposition to provide a detailed analysis of the shuttling process in a minimal [2]­rotaxane system across three solvents of varying polarity: DMSO, ACN, and CHCl_3_. Our results highlight the complex interplay between solvent–solute interactions, conformational constraints, and free-energy barriers, leading to the following key conclusions:

1. In less polar solvents such as CHCl_3_, the axle adopts a more compact conformation during shuttling, minimizing exposure to the environment and promoting intramolecular hydrogen bonding. This adaptability facilitates progression through enthalpically and entropically favorable intermediate states.
2. In CHCl_3_, the macrocycle shows a preferential localization at the distal station even at equilibrium, due to entropic stabilization and differential solvent exposure of the carboxylate group. This challenges common assumptions in kinetic modeling that rely on equal starting distributions and suggests a solvent-dependent directional bias may pre-exist prior to fuelling.
3. Solvent effects and intramolecular preorganization play a key role in the autonomous operation of the fuel-driven ratchet. While steric hindrance and hydrogen bonding have been proposed to explain reduced fuel reactivity when the ring is at the proximal station, we find that when it is at the distal one, despite the catalytic carboxylate appears more engaged in stabilizing interactions with nearby rotaxane components, the macrocycle may favors fuel-to-waste catalysis due to subtle structural organization. Specifically, coordination between the carboxylate, distal fumaramide, and macrocycle stabilizes a prereactive geometry that lowers activation barriers by reducing solvent reorganization and entropic costs. This mechanism offers an alternative to explanations based solely on nucleophilicity or accessibility.
4. Complementing the structural preorganization described above, our data also support a solvent-mediated contribution to the fuel-to-waste step. Beyond its role as a nucleophile, water may enhance ester electrophilicity through hydrogen bonding and local solvation effects, particularly when the macrocycle is positioned at the proximal station. In our simulations, this configuration results in higher solvent accessibility of the carboxylate group. These findings suggest that gating may arise not only from macrocycle-induced polarization, but also from differences in solvation environments dictated by ring position.
5. Across all tested solvents, the highest free-energy barriers associated with shuttling fall in the range of 9–13 kcal/mol, corresponding to time scales in the microsecond regime. However, the symmetry and ruggedness of the energy landscape vary: ACN features a smoother, more symmetric profile, while DMSO and CHCl_3_ exhibit multiple intermediates and directionally asymmetric barriers.

These findings highlight the critical role of environmental tuning in controlling the kinetics and directionality of molecular machines. The insights gained here may inform the rational design of next-generation autonomous rotaxanes, particularly those operating under nonequilibrium conditions or in complex solvent mixtures. Moreover, by clarifying the enthalpic and entropic factors contributing to the macrocycle’s shuttling, this study provides a framework for integrating thermodynamic landscapes with kinetic control in the operation of synthetic molecular devices.

## Supplementary Material



## Notes

Details on the procedures for the parametrization of the molecular models and on the simulations’ setup, along with additional simulation data (PDF)

## Abbreviations

ACN: acetonitrile
AMM: artificial molecular machine
CHCl_3_: chloroform
COM: center of mass
CV: collective variable
DMSO: dimethyl sulfoxide
FES: free energy surface
HOAt: 1-hydroxy-7-azabenzotriazole
HOBt: hydroxybenzotriazole
MD: molecular dynamics
MIM: mechanically interlocked molecule
NEB: nudged elastic band
WT-MetaD: well-tempered metadynamics
