# Supplementary material for: Solvent-Driven Modulation of Shuttling Dynamics in an Autonomous Chemically Fueled Information Ratchet
Source: J Phys Chem B. 2025 Oct 15;129(43):11283–96. doi: 10.1021/acs.jpcb.5c05092 (PMC12581131; doi:10.1021/acs.jpcb.5c05092)
Supplement: Supplementary file 1 [file jp5c05092_si_001.pdf]

<u>**Supplementary Information for:**</u>

Solvent-Driven Modulation of Shuttling Dynamics in an Autonomous Chemically Fueled Information Ratchet

Giuseppe Silvestri†, Mattia P. Fossati†, Federica Arrigoni†, Luca Bertini†, Giuseppe Zampella†, Luca De Gioia† and Jacopo Vertemara†

†*Department of Biotechnology and Biosciences BtBs, University of Milano-Bicocca, Piazza dell'Ateneo Nuovo 1, 20126 Milan, Italy*

E-mail: jacopo.vertemara@unimib.it, luca.degioia@unimib.it

Computational Details

Simulation Setup

As a first step, we built an all-atom model for the [2]rotaxane. The initial structure was derived from X-ray crystallography data (CCDC deposition code: 2191007), which features the macrocycle in a proximal position and a *t*-butyl ester derivative on the carboxylate moiety. After refining the structure, which involved removing residual solvent molecules and the *t*-butyl derivative, the model system was parameterized using the Generalized Amber Force Field 2 (GAFF2)[1,2]. Partial charges for the atomistic models were calculated using the RESP method[3], computed at the Hartree-Fock level of theory with the 6-31G** basis set, as implemented in *Gaussian16*[4]. The *AmberTools23* package[5] was utilized for system parameterization. The model was solvated with three explicit solvents DMSO, ACN, and $CHCl_3$

respectively, so that we could assess how solvent properties influence the dynamics of rotaxane. Solvent parameters were sourced from the virtualchemistry.org database[6,7]. A potassium ion was added to each simulation box to neutralize the negative charge of the carboxylate. To prevent electrostatic interactions between the ion and the carboxylate that could affect the proper rotaxane's conformational sampling, a potential wall was applied around the carboxylate, with a radius of 2 nm and a force constant of 2 kcal/mol·Å². All systems were minimized using the steepest-descent algorithm and then equilibrated under NPT conditions. Production runs were performed in the NPT ensemble for up to 1 µs. Temperature and pressure were kept at 300 K and 1 bar using the v-rescale thermostat[8] and the Parrinello-Rahman barostat[9]. Long-range electrostatic interactions were handled using the PME method[10] with a cutoff of 1.2 nm. Van der Waals interactions were truncated at 1.2 nm. The LINCS algorithm[11] was used to constrain all hydrogen-involving bonds, and the Leap-Frog integrator was used to propagate dynamics with a 2 fs time step. All simulations were carried out using GROMACS[12] 2022.3, with the system patched using PLUMED[13] version 2.8.1. Switching rates were computed via infrequent WT-MetaD, following the procedure established by Tiwary and Parrinello[14], which enables accurate estimation of transition times in rare-event processes.

System-specific simulation protocols

The following section outlines the simulation strategies employed for analysing the shuttling dynamics of each system under study. For each solvent environment, we tailored the parameters of the WT-MetaD to ensure both the convergence of the free-energy landscape and the reliability of kinetic observables. In particular, the bias factor in the standard WT-MetaD simulations was tuned based on the estimated free-energy barriers associated with the shuttling process. For infrequent WT-MetaD simulations, the Gaussian height and starting deposition frequency were further optimized to maximize the KS test p-value after Poisson fitting of the obtained empirical cumulative distribution functions (ECDFs), as per established protocols[15]. To characterize the translational motion of the macrocycle along the axle, we performed WT-MetaD simulations using two collective variables: the distance d_{CC}, defined as the separation between atoms C_{1p} and C_{1d}, and the coordinate d, which tracks the macrocycle position along the axle normalized by its maximum extension. The latter CV was computed by

projecting the distance between the center of mass of the four macrocycle nitrogens and atom C_{1p} onto the axis. The maximum extension $d_{CC,max}$ was set to 2.7 nm after monitoring d_{CC} during preliminary unbiased MD simulations of the rotaxane in DMSO. The macrocycle's conformational dynamics were further assessed by monitoring the torsional angle ϕ, previously validated as a sensitive descriptor of ring configurations (see Fig. 2A in the main paper for schematic CV definitions). For the WT-MetaD runs, a Gaussian bias was deposited every 500 steps (equivalent to 1 ps), with an initial height of 0.5 kJ/mol and widths of 0.04 nm and 0.08 nm for d and d_{CC}, respectively. A bias factor of 20 was employed across all systems. The simulation time ranged from a minimum of 3 µs up to 8 µs, allowing multiple shuttling events to be sampled within each trajectory (specifically, 3 µs for ACN, 7 µs for $CHCl_3$, and 8 µs for DMSO). To estimate the uncertainty associated with the computed free-energy profiles, we applied a block averaging approach.

To estimate transition rates between the identified free-energy minima, we conducted 20 independent infrequent WT-MetaD simulations starting from each basin. The same CVs were biased, and the target basin was defined based on the 2D-FES obtained from standard WT-MetaD simulations (see main text Fig. 2). All individual transition times and related metadynamics parameters are reported in Table S1. For consistency, initial Gaussian biases in infrequent WT-MetaD were deposited every 1 ps, with heights ranging from 0.5 to 2 kJ/mol. Widths of 0.04 nm for d and 0.08 nm for d_{CC}, and bias factors between 5 and 20, were used depending on the solvent. The frequency-adaptive biasing scheme[16] was activated, allowing the deposition frequency to decrease over time. This approach facilitated rapid filling of energy basins while minimizing bias deposition near transition state regions. PLUMED checks for convergence every 500 steps by verifying whether both CVs fall within the target basin, at which point the simulation is stopped. To quantify the uncertainty associated with the estimated transition time τ, we applied a bootstrap resampling protocol. Specifically, for each solvent and transition direction, subsets of size M (ranging from 6 to the full number of available times, in steps of 2) were randomly sampled with replacement, and the average τ was computed over 50 bootstrap replicas. The standard deviation across replicas was taken as the error estimate. This procedure was used not only to evaluate statistical uncertainty but also to assess the convergence of the rate estimates: the stability of the average τ as a function of increasing M was used as an internal diagnostic of sample sufficiency. Finally, we

note that due to the logarithmic dependence of the Eyring equation on τ, symmetric errors in the mean transition time lead to asymmetric uncertainties in the activation free energy $\Delta G^{\ddagger}$. Specifically, a given positive deviation in τ results in a smaller change in $\Delta G^{\ddagger}$ than an equivalent negative deviation, making the lower error bound on $\Delta G^{\ddagger}$ appear larger. This asymmetry becomes particularly pronounced when τ carries a high relative error.

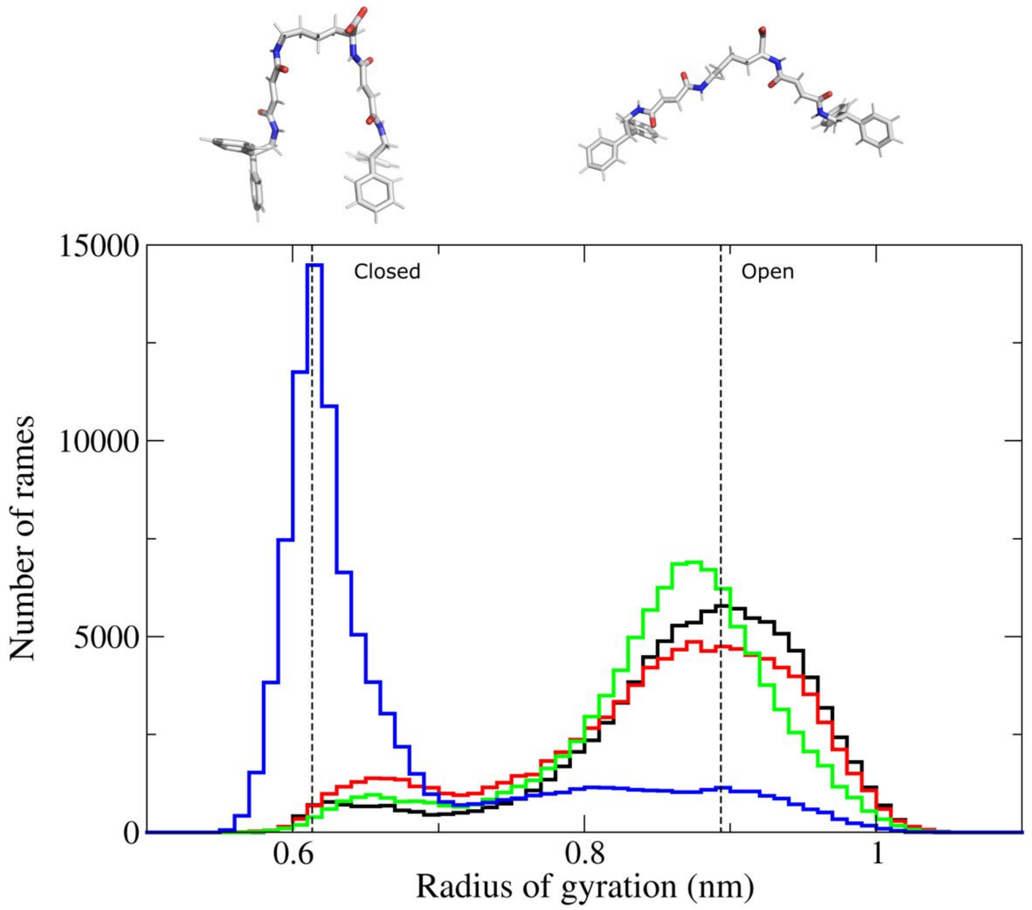

Figure S1: Radius of gyration of the rotaxane axle. The graph shows the distribution of radius of gyration values in the different solvents: black is DMSO, red is ACN, green is $CHCl_3$ and blue is water. The dashed lines represent the radius of gyration values for the open and closed conformations of the rotaxane axle, with the corresponding structure shown above each line.

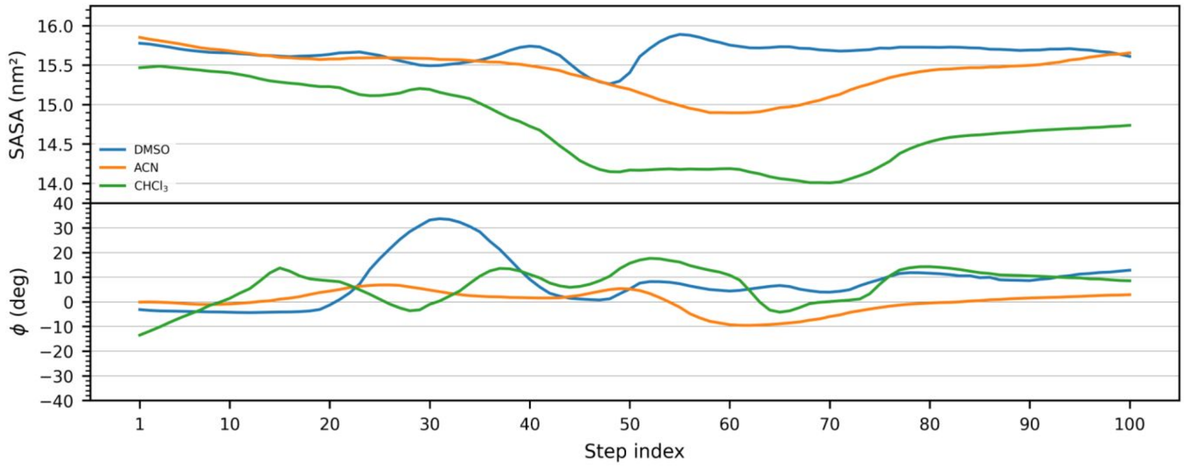

Figure S2: Solvent-dependent structural and chemical properties along the minimum free-energy pathway. Variation of solvent-accessible surface area (SASA, top plot) and macrocycle torsion angle ϕ (bottom plot) along the shuttling path in DMSO (blue), acetonitrile (orange), and chloroform (green). The x-axis indicates the progression along the minimum energy path (step index, from 1 to 100) connecting the two primary binding sites. In the top panel, SASA decreases notably in $CHCl_3$, indicating a stronger tendency of the rotaxane to adopt collapsed conformations in apolar environments. Conversely, DMSO maintains a consistently higher SASA, consistent with extended conformations stabilized by polar solvent interactions. In the bottom panel, the torsion angle φ reveals differences in macrocycle flexibility: while ACN exhibits minimal angular variation, DMSO and $CHCl_3$ show marked fluctuations, especially at mid-pathway regions, suggesting solvent-imposed structural rearrangements that affect the macrocycle conformation during shuttling.

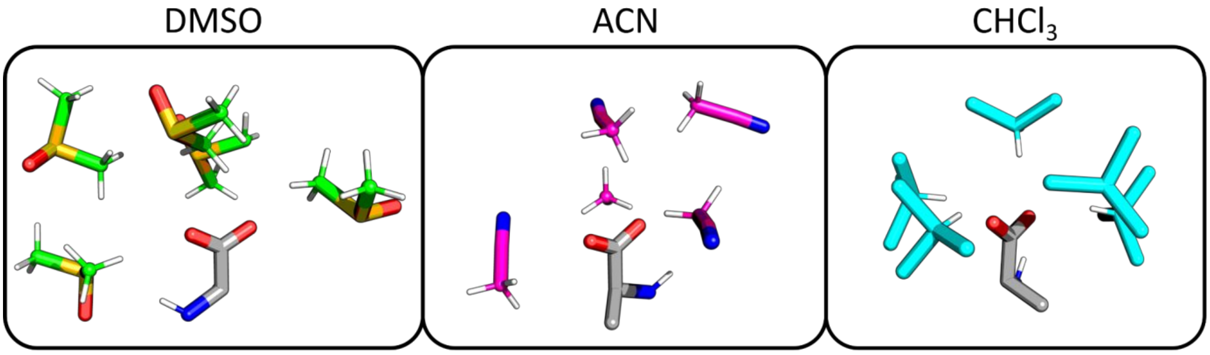

Figure S3: Representative snapshots of solvent–solute interactions involving the carboxylate group when the macrocycle resides at the proximal fumaramide station, shown for DMSO, ACN, and $CHCl_3$ (from left to right). In DMSO (left), the carboxylate forms multiple ion–dipole interactions with the methyl groups of surrounding solvent molecules (green). In ACN (center), solvation occurs primarily through dipole–ion interactions involving the methyl groups of acetonitrile, which orient toward the carboxylate but with structural. In $CHCl_3$ (right), weak but numerous $CH\cdots O^-$ hydrogen bonds are formed between the slightly polarized hydrogen atoms of chloroform and the carboxylate oxygens, generating a diffuse yet stabilizing interaction network.

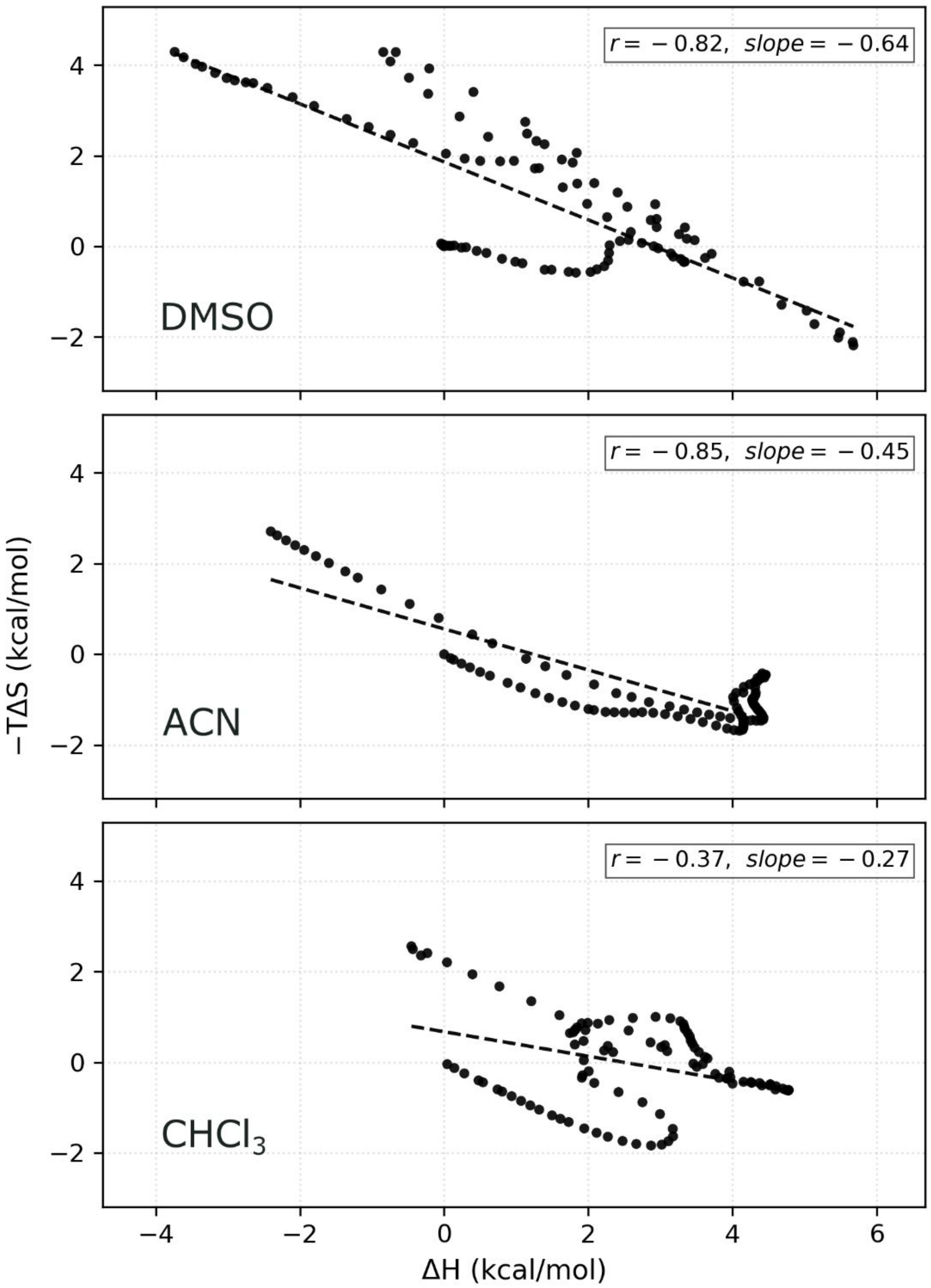

Figure S4: Scatter plot of -T Delta S versus Delta H in the three solvents. For each plot, the Pearson correlation coefficient and the slope of the regression line are reported.

Table S1: Setup and results of the infrequent WT-MetaD simulations used to estimate transition times between metastable states identified on the free-energy surfaces in DMSO, ACN, and $CHCl_3$. For each solvent, the table reports the transition studied, the collective variable ranges defining the target basin in *d* and d_{CC}, the initial Gaussian height and bias factor used during bias deposition, and the estimated transition time in seconds. Transition times were extracted from 20 independent infrequent WT-MetaD runs per transition using the protocol described in the section above.

	Transition	Target Basin *d* (nm)	Target Basin d_{CC} (nm)	Initial Bias Height (kJ/mol)	Bias Factor	Transition time (s)
**DMSO**	$A_1 \rightarrow C_1$	1.08 - 1.14	1.88 - 1.94	2	20	10^{-5}
**DMSO**	$C_1 \rightarrow A_1$	0.59 - 0.65	1.95 - 2.01	0.5	5	10^{-8}
**DMSO**	$C_1 \rightarrow D_1$	1.22 - 1.28	2.02 - 2.08	0.5	5	10^{-9}
**DMSO**	$D_1 \rightarrow C_1$	1.08 - 1.14	1.88 - 1.94	0.5	5	10^{-10}
**DMSO**	$D_1 \rightarrow E_1$	1.53 - 1.59	2.05 - 2.08	0.5	5	10^{-8}
**DMSO**	$E_1 \rightarrow D_1$	1.22 - 1.28	2.02 - 2.08	0.5	5	10^{-7}
**DMSO**	$E_1 \rightarrow F_1$	1.99 - 2.05	1.83 - 1.89	0.5	5	10^{-8}
**DMSO**	$F_1 \rightarrow E_1$	1.53 - 1.59	2.05 - 2.08	2	20	10^{-5}
**ACN**	$A_2 \rightarrow F_2$	2.01 - 2.07	1.84 - 1.9	2	20	10^{-4}
**ACN**	$F_2 \rightarrow A_2$	0.57 - 0.63	1.9 - 1.96	2	20	10^{-3}
**CHCl3**	$A_3 \rightarrow B_3$	0.69 - 0.75	1.77 - 1.83	0.5	5	10^{-9}
**CHCl3**	$B_3 \rightarrow A_3$	0.39 - 0.45	1.86 - 1.92	0.5	5	10^{-9}
**CHCl3**	$B_3 \rightarrow C_3$	0.97 - 1.03	1.61 - 1.67	0.5	5	10^{-9}
**CHCl3**	$C_3 \rightarrow B_3$	0.69 - 0.75	1.77 - 1.83	0.5	5	10^{-9}
**CHCl3**	$C_3 \rightarrow D_3$	1.36 - 1.42	1.41 - 1.47	0.5	5	10^{-9}
**CHCl3**	$D_3 \rightarrow C_3$	0.97 - 1.03	1.61 - 1.67	0.5	5	10^{-9}
**CHCl3**	$D_3 \rightarrow E_3$	1.94 - 2.00	1.22 - 1.28	2	20	10^{-6}
**CHCl3**	$E_3 \rightarrow D_3$	1.36 - 1.42	1.41 - 1.47	2	20	10^{-5}
**CHCl3**	$E_3 \rightarrow F_3$	2.09 - 2.15	1.72 - 1.78	0.5	5	10^{-8}
**CHCl3**	$F_3 \rightarrow E_3$	1.94 - 2.00	1.22 - 1.28	0.5	5	10^{-8}

References

(1) Wang, J.; Wolf, R. M.; Caldwell, J. W.; Kollman, P. A.; Case, D. A. Development and Testing of a General Amber Force Field. *J. Comput. Chem.* **2004**, *25* (9), 1157–1174. https://doi.org/10.1002/jcc.20035.
(2) He, X.; Man, V. H.; Yang, W.; Lee, T.-S.; Wang, J. A Fast and High-Quality Charge Model for the next Generation General AMBER Force Field. *J. Chem. Phys.* **2020**, *153* (11), 114502. https://doi.org/10.1063/5.0019056.
(3) Bayly, C. I.; Cieplak, P.; Cornell, W.; Kollman, P. A. A Well-Behaved Electrostatic Potential Based Method Using Charge Restraints for Deriving Atomic Charges: The RESP Model. *J. Phys. Chem.* **1993**, *97* (40), 10269–10280. https://doi.org/10.1021/j100142a004.
(4) Frisch, M. J. Gaussian 16 Revision C.01, 2016.
(5) Case, D. A.; Aktulga, H. M.; Belfon, K.; Cerutti, D. S.; Cisneros, G. A.; Cruzeiro, V. W. D.; Forouzesh, N.; Giese, T. J.; Götz, A. W.; Gohlke, H.; Izadi, S.; Kasavajhala, K.; Kaymak, M. C.; King, E.; Kurtzman, T.; Lee, T.-S.; Li, P.; Liu, J.; Luchko, T.; Luo, R.; Manathunga, M.; Machado, M. R.; Nguyen, H. M.; O'Hearn, K. A.; Onufriev, A. V.; Pan, F.; Pantano, S.; Qi, R.; Rahnamoun, A.; Risheh, A.; Schott-Verdugo, S.; Shajan, A.; Swails, J.; Wang, J.; Wei, H.; Wu, X.; Wu, Y.; Zhang, S.; Zhao, S.; Zhu, Q.; Cheatham, T. E. I.; Roe, D. R.; Roitberg, A.; Simmerling, C.; York, D. M.; Nagan, M. C.; Merz, K. M. Jr. AmberTools. *J. Chem. Inf. Model.* **2023**, *63* (20), 6183–6191. https://doi.org/10.1021/acs.jcim.3c01153.
(6) Caleman, C.; van Maaren, P. J.; Hong, M.; Hub, J. S.; Costa, L. T.; van der Spoel, D. Force Field Benchmark of Organic Liquids: Density, Enthalpy of Vaporization, Heat Capacities, Surface Tension, Isothermal Compressibility, Volumetric Expansion Coefficient, and Dielectric Constant. *J. Chem. Theory Comput.* **2012**, *8* (1), 61–74. https://doi.org/10.1021/ct200731v.
(7) Van Der Spoel, D.; Van Maaren, P. J.; Caleman, C. GROMACS Molecule & Liquid Database. *Bioinformatics* **2012**, *28* (5), 752–753. https://doi.org/10.1093/bioinformatics/bts020.
(8) Bussi, G.; Donadio, D.; Parrinello, M. Canonical Sampling through Velocity Rescaling. *J. Chem. Phys.* **2007**, *126* (1), 014101. https://doi.org/10.1063/1.2408420.
(9) Parrinello, M.; Rahman, A. Polymorphic Transitions in Single Crystals: A New Molecular Dynamics Method. *J. Appl. Phys.* **1981**, *52* (12), 7182–7190. https://doi.org/10.1063/1.328693.
(10) Darden, T.; York, D.; Pedersen, L. Particle Mesh Ewald: An N·log(N) Method for Ewald Sums in Large Systems. *J. Chem. Phys.* **1993**, *98* (12), 10089–10092. https://doi.org/10.1063/1.464397.
(11) Hess, B.; Bekker, H.; Berendsen, H. J. C.; Fraaije, J. G. E. M. LINCS: A Linear Constraint Solver for Molecular Simulations. *J. Comput. Chem.* **1997**, *18* (12), 1463–1472. https://doi.org/10.1002/(SICI)1096-987X(199709)18:12<1463::AID-JCC4>3.0.CO;2-H.
(12) Abraham, M. J.; Murtola, T.; Schulz, R.; Páll, S.; Smith, J. C.; Hess, B.; Lindahl, E. GROMACS: High Performance Molecular Simulations through Multi-Level Parallelism from Laptops to Supercomputers. *SoftwareX* **2015**, *1–2*, 19–25. https://doi.org/10.1016/j.softx.2015.06.001.
(13) Bonomi, M.; Bussi, G.; Camilloni, C.; Tribello, G. A.; Banáš, P.; Barducci, A.; Bernetti, M.; Bolhuis, P. G.; Bottaro, S.; Branduardi, D.; Capelli, R.; Carloni, P.; Ceriotti, M.; Cesari, A.; Chen, H.; Chen, W.; Colizzi, F.; De, S.; De La Pierre, M.; Donadio, D.; Drobot, V.; Ensing, B.; Ferguson, A. L.; Filizola, M.; Fraser, J. S.; Fu, H.; Gasparotto, P.; Gervasio, F. L.; Giberti, F.; Gil-Ley, A.; Giorgino, T.; Heller, G. T.; Hocky, G. M.; Iannuzzi, M.; Invernizzi,

M.; Jelfs, K. E.; Jussupow, A.; Kirilin, E.; Laio, A.; Limongelli, V.; Lindorff-Larsen, K.; Löhr, T.; Marinelli, F.; Martin-Samos, L.; Masetti, M.; Meyer, R.; Michaelides, A.; Molteni, C.; Morishita, T.; Nava, M.; Paissoni, C.; Papaleo, E.; Parrinello, M.; Pfaendtner, J.; Piaggi, P.; Piccini, G.; Pietropaolo, A.; Pietrucci, F.; Pipolo, S.; Provasi, D.; Quigley, D.; Raiteri, P.; Raniolo, S.; Rydzewski, J.; Salvalaglio, M.; Sosso, G. C.; Spiwok, V.; Šponer, J.; Swenson, D. W. H.; Tiwary, P.; Valsson, O.; Vendruscolo, M.; Voth, G. A.; White, A.; The PLUMED consortium. Promoting Transparency and Reproducibility in Enhanced Molecular Simulations. *Nat. Methods* **2019**, *16* (8), 670–673. https://doi.org/10.1038/s41592-019-0506-8.
(14) Tiwary, P.; Parrinello, M. From Metadynamics to Dynamics. *Phys. Rev. Lett.* **2013**, *111* (23), 230602. https://doi.org/10.1103/PhysRevLett.111.230602.
(15) Salvalaglio, M.; Tiwary, P.; Parrinello, M. Assessing the Reliability of the Dynamics Reconstructed from Metadynamics. *J. Chem. Theory Comput.* **2014**, *10* (4), 1420–1425. https://doi.org/10.1021/ct500040r.
(16) Wang, Y.; Valsson, O.; Tiwary, P.; Parrinello, M.; Lindorff-Larsen, K. Frequency Adaptive Metadynamics for the Calculation of Rare-Event Kinetics. *J. Chem. Phys.* **2018**, *149* (7), 072309. https://doi.org/10.1063/1.5024679.
